# Turning science into health solutions: KEMRI’s challenges as Kenya’s health product pathfinder

**DOI:** 10.1186/1472-698X-10-S1-S10

**Published:** 2010-12-13

**Authors:** Ken Simiyu, Hassan Masum, Justin Chakma, Peter A Singer

**Affiliations:** 1 McLaughlin-Rotman Centre for Global Health, University Health Network and University of Toronto, 101 College Street Suite 406, Toronto ON, M5G 1L7, Canada

## Abstract

**Background:**

A traditional pathway for developing new health products begins with public research institutes generating new knowledge, and ends with the private sector translating this knowledge into new ventures. But while public research institutes are key drivers of basic research in sub-Saharan Africa, the private sector is inadequately prepared to commercialize ideas that emerge from these institutes, resulting in these institutes taking on the role of product development themselves to alleviate the local disease burden. In this article, the case study method is used to analyze the experience of one such public research institute: the Kenya Medical Research Institute (KEMRI).

**Discussion:**

Our analysis indicates that KEMRI’s product development efforts began modestly, and a manufacturing facility was constructed with a strategy for the facility’s product output which was not very successful. The intended products, HIV and Hepatitis B diagnostic kits, had a short product life cycle, and an abrupt change in regulatory requirements left KEMRI with an inactive facility. These problems were the result of poor innovation management capacity, variability in domestic markets, lack of capital to scale up technologies, and an institutional culture that lacked innovation as a priority.

However, KEMRI appears to have adapted by diversifying its product line to mitigate risk and ensure continued use of its manufacturing facility. It adopted an open innovation business model which linked it with investors, research partnerships, licensing opportunities, and revenue from contract manufacturing. Other activities that KEMRI has put in place over several years to enhance product development include the establishment of a marketing division, development of an institutional IP policy, and training of its scientists on innovation management.

**Summary:**

KEMRI faced many challenges in its attempt at health product development, including shifting markets, lack of infrastructure, inadequate financing, and weak human capital with respect to innovation. However, it overcame them through diversification, partnerships and changes in culture. The findings could have implications for other research institutes in Sub-Saharan Africa seeking to develop health products. Such institutes must analyze potential demand and uptake, yet be prepared to face the unexpected and develop appropriate risk-mitigating strategies.

## Background

A traditional pathway for developing new health products begins with public research institutes as generators of knowledge, and the private sector as the translator of the knowledge into actual ventures. In the developed world, for example, early product commercialization – defined as conversion of an idea or technology to a product or service that generates profits or has an impact on the lives of everyday people [1] – is driven by public research institutes and universities [2-4]. Notable health products commercialized this way include the Boyer–Cohen “gene-splicing” technique that launched the biotechnology industry, and diagnostic tests for breast cancer and osteoporosis [5].

In the developing world, health products developed in public institutes and then transferred to the private sector include the first effective meningitis B vaccine, developed at the Cuban Finlay Institute and licensed to GlaxoSmithKline [6], and the antimalarial drug arteether (a semi-synthetic artemisinin derivative), developed at India’s Central Drug Research Institute and then sold in dozens of countries. In sub-Saharan Africa, an example of such a transfer is the sickle cell drug Niprisan, developed by the Nigerian Institute of Pharmaceutical Research and licensed to Xechem (see the paper on Niprisan in this BMC series [7]). Another example is the appetite-suppressing *Hoodia* plant, whose active ingredient was patented and out-licensed by South Africa’s Council for Scientific and Industrial Research [8].

Many deaths due to disease continue to occur in sub-Saharan Africa, e.g. 90% of the 1 million malaria deaths and 40% of the 1.3 million TB deaths globally in 2008 were in sub-Saharan Africa [9,10]. Yet less than 10% of global health R&D expenditures go toward developing solutions for the developing world, (commonly termed the 10/90 gap) [11]. This has led to calls for countries in sub–Saharan Africa to develop their own products as a way of addressing local health problems [12,13]. Since public research institutes are still the key drivers of basic research in sub-Saharan Africa [14,15], and the private sector is inadequately prepared to commercialize ideas that emerge from these institutes [16], public research institutes continue to be important vehicles for developing health solutions in the African context.

These issues are illustrated by the experiences of the Kenya Medical Research Institute (KEMRI). KEMRI is one of Africa’s premier health research institutions, and successfully advised Kenya’s government to withdraw the anti-malaria drugs Chloroquine and Daraprim. KEMRI is also one of the very few research institutes in Africa that has attempted to commercialize its own technologies.

We used a case study design. Our analysis is based on semi-structured interviews and on-site observations that took place at KEMRI’s Nairobi and Kisumu campuses during the months of June and July 2008, and subsequent visits and interactions over 2008 and 2009 as well as secondary analysis of peer-reviewed literature, articles, news items and web sites. We conducted interviews with informed consent with personnel of KEMRI’s management, product manufacturing facility, scientific staff, and IP management team. Representatives of KEMRI were asked to fact-check the case study; the analysis and interpretation is our own. All quotes are from the interviews unless otherwise noted, and with permission. This study was approved by the Office of Research Ethics of the University of Toronto.

In this article, we explore the role of public research institutes in health product development and commercialization by examining the case of KEMRI. We begin by describing the history of health product development at KEMRI, discuss its successes and challenges, and suggest lessons for policy makers, donors, institutional managers, and African scientists.

## Discussion

### Context

In 1979, two years after the breakup of the East African Community, the Kenya government established KEMRI as a state parastatal (government-owned organization) to fill the health research void caused by the dissolution of the East African Medical Research Council. Its vision is to be a leading centre of excellence in the promotion of quality health, with a mission of improving the quality of health and human life through research.

Beginning with a staff of five research scientists located in one centre in Nairobi in 1979, KEMRI has now grown to over 450 scientists. (See Table 1 for a timeline of KEMRI.) KEMRI has attempted to commercialize its own technologies by constructing a manufacturing facility for diagnostic kits for Hepatitis B (Hepcell) and HIV (Kemcom). Constructed in 2006, the production unit had about 20 employees, out of KEMRI’s total staff population (including non-scientists) in 2009 of roughly 1250. (Note that when we discuss KEMRI’s product development experience in this paper, it is often this production unit and allied units that are key players, as parts of a much larger organization.)

**Table 1:** KEMRI timeline

**1973**	East African Medical Research Institute established (EAMRI)
**1979**	Kenya Medical Research Institute is formed following dissolution of EAMRI
**1990**	KEMRON controversy
**1998**	Japanese International Cooperation Agency (JICA) with KEMRI scientists begins research into diagnostic kits
**2004**	KEMRI and Japanese scientists develop Hepcell and Kemcom.
**2005**	Construction of production facility for point of care diagnostics begins
**2006**	Linkage with Bridgeworks Africa to develop institutional IP policy
**2007**	Completion of production facility
**2008**	Launch of TBcide, a chlorine-based decontaminant for handling *Mycobacterium*.

The Institute has two units and eight research centers located in Nairobi, while an additional two centers are located in Kisumu and Kilifi respectively. KEMRI’s most recent ten year strategic plan estimated the annual budget at $37.5 million. The sources of funding are as follows; 50% from the Kenyan government, 45% from collaborating research partners such as major partner Wellcome Trust, and about 5% from internally generated funds that include clinical work, diagnostics, and academic services that KEMRI provides for clients [17]. Because of resource limitations, there is pressure on KEMRI to move from a non-profit operational model to one where an increasing share of operations is self-financed.

KEMRI is internationally known for participating in various programs including clinical research, AIDS vaccine development, and conducting clinical trials since 2003 for IAVI (the International AIDS Vaccine Initiative) [18]. It participated in a pan-African research project (MARA/AMRA) started in 1996 to map malaria risk and endemicity, which led to the development of ‘risk maps’ used in malaria control policy activities and geographical modeling of malaria, enabling the first accurate assessment of the disease burden in Kenya [19]. In collaboration with other African research institutes, KEMRI has reportedly also screened thousands of small molecules and natural products against schistosomiasis, malaria, and tuberculosis through the Helminth Drug Initiative, helping provide impetus for the new African Network for Drugs and Diagnostics Innovation [20].

KEMRI’s commercialization initiatives can be traced back to August 24, 1990. On this date, KEMRI announced that it had discovered a cure for HIV/AIDS called KEMRON^®^[21]. Several groups around the globe that had been involved in developing the product were reported to be jostling for ownership and distribution rights [22]. Controversy also arose, with claims that KEMRON^®^ had undergone uncontrolled clinical trials and that further testing would be required to validate its effectiveness [23]. In the US, the case of KEMRON became political with groups alleging that it was a deliberate attempt to stifle African innovation [24,25]. While this controversy was raging, a new drug called Immunoplex-N^®^ was patented for the management of AIDS [26]. KEMRI scientists interviewed believed that Immunoplex-N^®^, now available in numerous countries, has the same molecule as KEMRON^®^.

According to one of the scientists interviewed, all this happened because the institute lacked experience in innovation management and at that time had no intellectual property policy. The resultant controversy impeded further interest at KEMRI in research commercialization.

### The early years of product development

The idea to enter product development resurfaced in the late 1990s, prompted mainly by falling budget support by the government and donors, as well as by the desire to make an impact on society by producing health products suitable for local conditions. “Prior to that we were just a typical health research institute focusing on clinical and basic biomedical research,” says Dr. Gerald Mkoji, KEMRI’s Deputy Director. A Centre for Biotechnology, Research and Development was therefore established which was to focus on product development.

Early product development related activities included evaluating products developed in other countries to assess their quality and suitability for the Kenyan market. From KEMRI’s inception, Kenyan health product regulatory authorities like the Kenya Bureau of Standards, the National Public Health Laboratories, and the Pharmacy and Poisons Board would refer some drugs and diagnostics to KEMRI laboratories for evaluation, to determine their safety and efficacy prior to registration on the Kenyan market. According to Dr. Mkoji, KEMRI scientists soon realized that this focus on efficacy and safety was too narrow for effective delivery of health care products to Kenya’s population. Products could be safe and effective, but not suited to meeting local public health challenges – their cost might be too high, or they might require the use of electricity or additional technology that was not readily available in rural areas.

Consequently, according to Dr. Mkoji, KEMRI scientists began researching how to adapt products that had been shown to be effective in other countries, but were not well-suited for local conditions in their existing form. Such research focused initially on diagnostics, though there was also interest in pharmaceutical drugs. In the process, the scientists acquired skills applicable to full product development, including assessing market suitability and requirements.

### Technology Transfer from Japan

According to our interviewees, the next step towards product development came with the construction of a manufacturing facility. For 25 years, the Japan International Cooperation Agency (JICA) and KEMRI had collaborated in areas of parasitic and infectious disease research. The goal of the collaboration was technology transfer from Japan to Kenya through training and product development. One of the outcomes of this collaboration was the development of diagnostic kits. Prototype blood screening kits were developed in Japan, and then evaluated at KEMRI and adapted to Kenya. After ten years of product development and evaluation, the partnership led to the development of a Hepatitis B diagnostic kit (Hepcell) for testing blood for transfusion. Hepcell is based on reverse passive haemagglutination technology. This technology was ideal for the Kenyan market, as it was cheaper than transporting blood to laboratories for screening. It was also portable, and could be used in remote environments where there was no electrical power.

For three years, the kits were produced at a small scale in KEMRI’s laboratory using locally available raw materials including cell lines, thus making them cheaper. By 2004, KEMRI had broadened its objectives and began thinking of moving into commercial production by constructing a product manufacturing facility at the institute. “We started thinking of how we could start producing our own innovations and moving products to the market at affordable costs,” says Dr. Wesley Ronoh, marketing manager.

In order to scale up, a US$5-million manufacturing facility was constructed. The decision to construct a manufacturing facility instead of licensing out manufacturing was taken since no manufacturing facilities that could be licensed to existed locally. The facility was funded with a grant from JICA (the Japan International Cooperation Agency) and additional funding from the Government of Kenya. This state-of-the-art facility opened in 2007 and hosts a current Good Manufacturing Practice (cGMP) certified production unit, laboratories, and offices, all with the goal of pushing diagnostic products to market. The facility is equipped with ultracentrifuges, high capacity centrifuges, a lyophilizer, a precision spraying machine, a precision guillotine machine, a high capacity incubator, and an animal house.

### A white elephant?

The manufacturing facility was designed to produce two types of diagnostic kits: Hepcell kits for detection of hepatitis B virus, and Kemcom kits for detection of HIV in the blood (both based on particle agglutination technology). However, the management of KEMRI soon realized that there was no institutional mechanism to engage in commercialization activities. Dr. James Kimotho was then hired as a Production Manager to help the institution manage the facility and its innovations. “We then began to think about intellectual property [and] commercializing innovations, because after putting in place the infrastructure, it became necessary to look and think about moving products to the market at the lowest price and producing our innovations. As a result of that, we set up a marketing office and a technology transfer office, which now began to put in place the necessary institutional policies for commercialization,” says Dr. Ronoh.

But in 2008, KEMRI experienced an unexpected setback: the Kenyan government, a major customer for the diagnostic kits produced at the unit, suddenly stopped purchasing the kits. This was as a result of global World Health Organization (WHO) recommended changes on the screening of blood. WHO recommended use of ELISA based technologies and at the same time discouraged use of particle agglutination based technologies for screening blood in blood transfusion centres. This resulted in blood screening in Kenya moving from using particle agglutination technologies to only ELISA based technologies that were centralized in 5 blood transfusion centres in the country. The health clinics would no longer be supplied with KEMRI’s Hepatitis B and HIV diagnostic kits. In the opinion of Dr. Ronoh, “The institute suddenly found itself with a white elephant – that is, it had expensive and excellent facilities that were not being utilized.”

This setback required a new strategy on how best to use the facility. One approach was to produce point of care diagnostics in the form of rapid Hepatitis B & C and HIV test kits, which are suitable for blood screening and diagnosis. In contrast to KEMRI’s earlier kits, these rapid test kits were based on immunochromatographic technology, and were suitable for adoption as point of care tests. Prior to the development of domestic kits, such kits were imported from Japan, Europe, and other sources by the Ministry of Health. With help from JICA, technology was transferred to KEMRI. KEMRI scientists then developed these rapid test kits, and by late 2008 these new kits were being produced at the facility. However, the facility is still underutilized. The plant’s current annual revenue is still minimal at about USD $100 000, while its expenses are estimated to be around USD $50 000. According to Dr. Ronoh, the potential market is promising as Kenya requires about 2.4 million HIV rapid test kits a year for use in Voluntary Counseling and Testing centres (VCTs); if the government was to approve the use of these kits, KEMRI could earn at least one million dollars per year.

### Technology assessment and institutional IP policy

Dr. Ronoh reported that Bridgeworks Africa (a local venture capital firm) was involved in technology assessment of potential products. According to him, the first products were selected on the basis of their market potential, and included a plant extract for multi-drug resistant TB; a plant extract for topical application in the management of Herpes Zoster; a plant extract for treatment of sleeping sickness; and an existing drug used for treating cancer which has also shown efficacy against malaria.

The relationship with Bridgeworks was based on a confidentiality and non-disclosure agreement negotiated on a case by case basis with right of first refusal going to Bridgeworks. Dr. Ronoh reported that due to restructuring at Bridgeworks, this process had stalled around 2008, and that KEMRI had its own plans to develop an institutional IP tracking system to track its technologies.

According to Dr. Ronoh, Bridgeworks Africa and the Kenya Intellectual Property Institute (KIPI) assisted in the development of an institutional IP policy, and in establishing relationships which resulted in training programs at international organizations involved in innovation management like the Swedish government innovation agency (VINNOVA) and the Office of Technology Transfer of the NIH in the US. Other relationships were with pharmaceutical companies based in Kenya for product distribution, and with the Kenya Industrial Research and Development Institute (KIRDI) which is Kenya’s national incubator. KEMRI also recruited a legal officer whose duties include negotiating contracts with partners who want to invest in KEMRI products.

### Product diversification

After the government stopped buying from KEMRI in 2008, KEMRI began to diversify its product portfolio by instituting changes on how to approach commercialization. This was done by opening up activities of the marketing department to other areas instead of focusing only on diagnostics, which resulted in the development of other products.

TBcide^®^ is a standardized chlorine-based decontaminant claimed to prevent occupational exposure when handling Mycobacterium-infected surfaces. According to the scientist who developed TBcide^®^, it eliminates residual mycobacterium pathogens from surfaces, unlike many conventional disinfectants [27]; it also has the advantage of being cheaper than imported disinfectants. The product was tested in hospitals in Kenya and patented in 2008 through the Kenya Intellectual Property Institute (KIPI) [28]. In a first for KEMRI, production was outsourced. According to Dr. Ronoh, a private firm was identified to produce TBcide^®^ and market the product on behalf of KEMRI. He stated that the firm will use KEMRI’s appointed distributor and receive marketing support from KEMRI.

Another product being developed at KEMRI is KEMTAQ, which contains a heat-stable DNA TAQ polymerase that was first isolated from the bacterium *Thermus aquaticus* but is now produced using DNA recombinant technology. It is used in Polymerase Chain Reaction (PCR) to amplify DNA to adequate quantities that can be handled or quantified easily. DNA TAQ polymerase is a cornerstone of molecular diagnostic techniques. The technology is patented elsewhere but not in Kenya [29]. The process technology for production has been adapted to local conditions using plasmids and *E. coli* for expressing TAQ polymerase that KEMRI scientists have been able to reproduce using recombinant technology. It is now being supplied to laboratories in Kenya. All the above products are listed for ordering online from KEMRI [30].

KEMRI scientists are working with traditional healers to develop antimalarials isolated from plants at KEMRI’s Centre for Traditional Medicine and Drug Research [31]. Dr. Jennifer Orwa, Principal Scientist at KEMRI, indicated that partnerships have been formed with local communities for information and supply of these traditional plant technologies. Memorandums of Understanding have been signed by both traditional healers and institute scientists, which clearly state prior informed consent and benefit sharing.

### Future plans

According to Dr. Ronoh, KEMRI has been approached for contract manufacturing by domestic and international companies that want to develop health products for low resource settings – reportedly due to its location in a tropical country, and facility availability for trial production of diagnostic technologies. Negotiations are ongoing with several of these organizations to use the facility to produce locally adapted diagnostic kits for the African market.

Dr. Mkoji revealed that one of KEMRI’s potential strategies is to convert the marketing division into an independent enterprise, without abandoning the overall objective of targeting the poor. Additional funding for product development might come from income generating activities of other departments like diagnostic services, clinical services, analytical services, training, and student attachments. Proceeds from these activities would then be used to support innovation. KEMRI plans to retain control—and equity—over products by manufacturing most of them partially or fully in its own production facility.

KEMRI’s marketing department hopes to compete internationally, especially in regional economies, by producing products that can compete globally. “The first step is to get WHO accreditation for our manufacturing facility and products, which we are pursuing,” says Dr. Ronoh. At the same time, KEMRI is trying to influence government policy towards health innovation by recommending to the Kenyan government the need to sensitize implementing officers on the importance of supporting domestic health innovation, by means such as national procurement strategies for suitable health products.

In June 2010, the U.S. President's Emergency Plan for AIDS Relief (PEPFAR) reportedly began a business and strategic assessment of the KEMRI Production Facility, to evaluate its procurement potential as a supplier of HIV diagnostics [32].

We turn now to an analysis of KEMRI’s challenges and business model, and the lessons KEMRI may hold for future development of health solutions in low-resource settings.

### Barriers to commercialization

Health product innovation does not occur in isolation, and in sub-Saharan Africa is fraught with many challenges and barriers that arise from within and outside the institution [33]. Many of these challenges are a result of weaknesses in the national innovation system of the country.

Our analysis revealed that one major challenge for KEMRI is crossing the “valley of death”: the resource gap between R&D activities and commercialization [34]. The absence of private sector investments in biotechnology and lack of venture capital in Kenya have negatively affected the chances of most of KEMRI’s R&D being commercialized. Dr. Peshu, Director, Centre for Geographic Medicine and Research Coast, Kilifi, summarized it as follows, “We have quite a number of products that have shown activity *in vitro*, in the lab, but now the next stage is to demonstrate more evidence that the products work. The donors are not willing to risk their research grants, for example, for *in vivo* studies in animals – and then, if this succeeds, to clinical trials. They only support lab studies, so there's a valley of death so to speak.”

The lack of a national policy on health innovation in Kenya was mentioned by interviewees as a factor that has hampered product development and commercialization at KEMRI. The overreliance of the government on donor agencies to fund the Ministry of Health as well as supply it with imported health products does not encourage domestic innovation. The government of Kenya does not protect local innovators from foreign competition, unlike countries like Japan and emerging economies like India and China [35,36]. According to Dr. Ronoh, when approached to purchase from KEMRI, foreign governments (especially those within East Africa) and investors question why the Kenyan government itself is not buying from KEMRI. Part of the problem, says Dr. James Kimotho, “is that the health sector in Kenya is classified in the social pillar and not in the economic pillar in government development policy documents, like Vision 2030”. It is therefore not seen as a potential revenue earner, but as a sector that drains exchequer funds.

Another barrier to product commercialization was the culture of “publish or perish” among scientists. One scientist remarked that promotions were pegged to the number of publications a researcher produced, regardless of their relevance to clinical applications or solving health problems. This has hindered innovation, as researchers focused primarily on basic scientific research. Dr. Ronoh and the staff at the marketing office began efforts to change the mindset of the scientists, with the support of training programs offered by Bridgeworks Africa [37]. One weakness identified was that local universities did not incorporate intellectual property knowledge as part of their curriculum for training scientists.

A final barrier mentioned during the interviews is managing intellectual property. Despite KEMRI having its own institutional IP policy and training for scientists, these efforts have been weakened by existing collaborative agreements with research partners that do not adequately address IP issues. According to Dr. Ronoh, research collaboration partners have often maintained clauses entitling them to any IP that comes from a research project that they have funded. In addition, he reports that some KEMRI scientists have negotiated collaborative arrangements with other institutions and groups without paying much attention to IP issues, with the resulting risk that technologies developed from such collaborations may end up in foreign countries without benefiting KEMRI scientists. He says that since the adoption of the institutional IP policy, establishment of the IP office, and recruitment of a legal officer, all collaborative arrangements and agreements are required to include a section that addresses IP issues.

### Implicit open innovation business model

KEMRI does not have an explicit business model. However, based on our analysis of the interviews, it appears that the strategy that has evolved to overcome barriers to commercialization closely resembles an open innovation business model [38]. This model assumes that an organization can and should use external ideas as well as internal ideas, and external as well as internal paths to innovation and market – all while trying to advance its technology. If an organization lacks internal product development capabilities, then it can leverage external resources through a combination of strategies. Conversely, an organization can maximize use of its internal production capabilities by attracting innovations and ideas that have been developed outside the organization, and fully developing them.

In the case of KEMRI, this open innovation business model has three aspects:

#### Linking with investors

From our interviews, the management of the marketing department of KEMRI realized they had limited internal capacity in product commercialization, especially in drug production, and turned to outside organizations for help. KEMRI began exploring outsourcing of some production functions in 2008 with TBcide^®^, as described above. Dr. Ronoh states that KEMRI staff have conducted a GMP audit of local pharmaceutical manufacturers so that only GMP compliant organizations will be selected as partners to produce products on behalf of KEMRI. The investment from JICA was instrumental in developing the original manufacturing facility for diagnostic kits, and opportunities for contract manufacturing may bring in new investors in the future.

#### Technology assessment

In order to make good business decisions, it is necessary for an institution to know the potential of the technologies it has. KEMRI pursued technology assessment of potential products in partnership with Bridgeworks Africa. It subsequently formed its own plans to develop an institutional IP tracking system to track its technologies.

#### Managing IP

As discussed above, KEMRI developed an institutional IP policy and strengthened innovation management capabilities in partnership with a range of external collaborators, both African and international. According to Dr. Kimotho, developing an institutional IP policy is the first step to engaging external investors, and the ability to negotiate licenses and contracts is critical if an institution is to maximize benefits and prevent the theft of IP. He says, “you can't go into product development with naiveté. You will be ripped off.” Interviews with other marketing department personnel indicated that their department aims to ensure that technologies developed are patented before discussions are held with potential investors.

### Lessons learned

Our analysis of KEMRI’s experiment with product development suggests several lessons that may be beneficial for other institutions in sub-Saharan Africa that are grappling with similar challenges.

#### Investments in research need to be accompanied by investments in innovation management

As the case of KEMRI has shown, institutions should develop their own capacity in innovation management. “Inventing is one thing. Taking an invention to market is completely different,” says Dr. Kimotho. Proper integration of innovation management allows organizations to validate the science, establish and integrate technologies, prevent loss of IP, and strengthen business models. KEMRI is developing its capacity in innovation management by sending its staff to participate in local and international training programs.

#### Focusing on local markets can generate health benefits, and rapidly create revenue

Health products that KEMRI has focused on so far are point-of-care diagnostics, traditional medicines, and disinfectants. Most technologies are customized to address domestic health problems such as malaria, tuberculosis, HIV, and other neglected diseases. Advantages of local technologies include their low cost, convenience, and adaptability to local settings. Both Hepcell and Kemcom were low cost solutions that were portable, cheap, and did not require electricity. This strategy has been a source of revenue and a morale booster to the scientists. Because product development takes a long time and most public research institutes in the developing world face significant shortfalls in finance, it is important to target areas that will quickly bring in revenues that can be ploughed back into new products. These revenues can also be used to purchase more modern equipment that the organization requires, and to demonstrate research impact. If sales of health technologies result in the marketing unit becoming self-sustaining, this may sway policy makers and management to develop favorable policies. KEMRI management is considering making the production unit autonomous, so that revenues from it do not have to go to the central institutional pool, but instead can be invested back into the facility.

#### Proper strategic planning and risk mitigation strategies are critical in volatile business environments

The importance of proper planning in product development and the ability to adapt to different scenarios cannot be over-emphasized. Product life cycles can be short. In addition, product development in the developing world occurs in highly uncertain environments [39]. KEMRI faced near disaster after building its production facility because it implicitly assumed that there would be a guaranteed market. Its experience suggests the value of engaging with relevant regulatory bodies, and the value for both governments and research institutions of supporting strategic local production. Commercialization strategies need to be incorporated when a product is still being researched. Proper planning will ensure that arrangements are in place in case of unforeseen changes, in both technology and markets. Planning includes diversification of the product portfolio: KEMRI diversified from development of diagnostics to development of disinfectants against Mycobacterium and enzymes for DNA amplification.

#### Public research institutes should be prepared for a long and difficult process to make health product development work

Research institutes must understand that commercialization by public institutes is a complex and difficult process involving many players. While reductions in government funds and fierce competition for donor grants have prompted institutes like KEMRI to look for other sources of revenue, its experience with product development has not been an easy alternative. In addition, the intricacies involved in balancing between a public institute’s social mandate and making profit through developing products can be tricky. “It is very difficult to produce products for the poor” says Dr. Mkoji. “You have to recover your costs and make some little profit, yet you must make products that are affordable, which is difficult”, he adds. Institutes therefore need to understand that commercialization is laborious, and they should be prepared to be in it for the long haul.

#### Imitation through technology transfer can be the first step to fostering innovation

Technological capabilities are critical to product development [40]. Local capacity in innovation can be developed through technology transfer, by first establishing facilities that produce variants of products from the developed world. Skills and techniques acquired in these facilities can then be adapted to similar areas. An example of this method of domestic skill development was in Japan where organizations developed their industrial capabilities through imitation of foreign technology and reverse engineering [41]. Local production of Hepcell and Kemcom diagnostic kits enabled scientists to acquire skills that could be applied to other diagnostics.

#### Research institutes should adopt open business models

Literature on technology management shows that organizations have difficulty managing innovations that they have no previous experience developing [42]. KEMRI’s strengths include R&D capacity and capabilities in developing diagnostics, but it is weaker in other areas such as drug development. KEMRI has pursued a business model that is closely aligned to an open business model [43], whereby it has tried to commercialize its own internal technologies, but sought external linkages and assistance where it lacked capacity. “We have tried to leverage our strengths, especially in diagnostics development, by looking for technologies outside – and tried to use internal mechanisms to develop them” says Dr. Ronoh. To solve local health problems, research institutes need to leverage their strengths while acknowledging their weaknesses. KEMRI has approached this through linking with investors, outsourcing and licensing, and contract manufacturing.

## Summary

This case study of KEMRI reveals the dynamic interaction between institutional factors, external factors, and product development in a public research institute in sub-Saharan Africa. In the early years, KEMRI’s experiment with product development was not very successful, with two key reasons being lack of innovation management experience and lack of an institutional IP policy. Later, KEMRI improved its innovation management and technological capabilities through recruitment and training, but these were still inadequate to ensure the success of its next effort in product development.

Major barriers that KEMRI faced included shifting markets, lack of scientific and production infrastructure, inadequate financing for product development, and weak human capital with respect to innovation. Reliance on a limited set of products, i.e diagnostics, proved to be dangerous as that product line relied on government purchasing. Shortly after construction of a production unit, the government stopped purchasing diagnostics from KEMRI, resulting in an underutilized facility.

KEMRI has since diversified its product portfolio. This has led to limited success in product development, with a portfolio which now includes a disinfectant and rapid HIV and Hepatitis B test kits. Given the weakness of internal capabilities in some areas of product development, KEMRI has adopted an open innovation business model which includes partnerships with other firms. Internal changes have also been instituted, including development of an institutional IP policy and programs promoting a culture of innovation among scientists.

Lessons were identified in the study that could have implications for other research institutes in Sub-Saharan Africa seeking to develop health products. These lessons include: investments in research need to be accompanied by investments in innovation management; institutions may wish to initially focus on local markets to generate immediate health and financial benefits; a volatile business environment in Africa implies a need for proper strategic planning; and open business models can help institutions leverage outside strengths to develop products. Expert demand analysis and forecasting is also critical.

KEMRI’s experience shows that with the right policies, challenges to product development are not insurmountable. Research institutes in Africa can turn science into health solutions for local health problems, thus reducing Africa’s health burden.

## Competing interests

Wesley Ronoh has consulted for the McLaughlin-Rotman Centre for Global Health.

## Authors' contributions

KS, HM, JC, and PAS contributed to the concept and design of this study, analyzed the findings, and participated in manuscript development. KS and PAS participated in site visits.
